# Removal of Methylene Blue from Aqueous Solutions Using a New Natural Lignocellulosic Adsorbent—Raspberry (*Rubus idaeus*) Leaves Powder

**DOI:** 10.3390/polym14101966

**Published:** 2022-05-11

**Authors:** Giannin Mosoarca, Simona Popa, Cosmin Vancea, Mircea Dan, Sorina Boran

**Affiliations:** Faculty of Industrial Chemistry and Environmental Engineering, Politehnica University Timisoara, Bd. V. Parvan No. 6, 300223 Timisoara, Romania; giannin.mosoarca@upt.ro (G.M.); sorina.boran@upt.ro (S.B.)

**Keywords:** lignocellulosic adsorbent, methylene blue, adsorption, isotherm, kinetics, Taguchi optimization

## Abstract

In this work, raspberry (*Rubus idaeus*) leaves were converted to powder and used as a new natural lignocellulosic low-cost adsorbent for methylene blue removal from aqueous solutions. Different techniques (FTIR, SEM, color analysis, and pH_PZC_ determination) were applied for adsorbent characterization. The effects of pH, ionic strength, contact time, adsorbent dose, initial deconcentration, and temperature on adsorption capacity were investigated. Equilibrium, kinetic, and thermodynamic studies have shown that the adsorption is best described by the Sips isotherm and pseudo-second-order kinetic model and that the process is spontaneous, favorable, and endothermic, involving physisorption as the main mechanism. The maximum adsorption capacity was 244.6 (mg g^−1^) higher compared to other adsorbents based on plant leaves. The Taguchi method and the ANOVA analysis were used to optimize the adsorption conditions. The contact time was the factor with the highest influence on the process, while the temperature had the lowest influence. A desorption study was also performed to determine the possibility of adsorbent regeneration.

## 1. Introduction

Anthropogenic activity is the main cause of environmental pollution and, implicitly, water contamination. Dyes from residual effluents can cause severe adverse toxicological and aesthetic effects on the aquatic environment [1,2,3,4,5].

Nowadays, methylene blue is one of the most widely used dyes in various anthropogenic activities. It has numerous applications in the textile, tannery, plastics, food, cosmetics, and paper industries. It is also used in the pharmaceutical industry and medical practice (methaemoglobinemia treatment, cyanide poisoning treatment, or as a staining agent) [1,2,3,4,5,6,7,8,9]. But this dye can also cause various negative effects on human health and the environment. Adverse effects of this dye include the following: skin, eye, mouth, throat, and stomach irritation, respiratory problems, vomiting, diarrhea, dyspnea, tachycardia, and high blood pressure [2,8,9,10].

In order to remove the dyes (including methylene blue) from aqueous solutions, several different techniques have been applied as follows: coagulation, flocculation, precipitation, membrane processes, chemical oxidation, electrochemical processes, catalytic reduction, photocatalytic degradation, ion exchange, adsorption, microorganism degradation, membrane bioreactors, aerobic, and anaerobic biological treatments [1,2,4,5,6,7,8,9,10,11,12,13,14,15,16,17,18,19,20].

Adsorption has proven to be a simple, efficient, and economical method that can use a wide variety of synthetic and natural materials as adsorbents [1,2,3,4,5,6,7,8,21,22,23]. The adsorbent price is a very important factor when deciding the practical applicability of a new adsorbent material. An absorbent is considered “low cost” if it is found in large quantities and needs minimal processing before use or if it is a by-product or waste from another activity [1,4,6,7,8,12,16,17]. Thus, various natural materials (clay-minerals, zeolites), bio-adsorbents (algal, bacterial, fungal, various biomasses), and agricultural or industrial wastes (fibers, peals, leaves, barks, fly ashes, sludges) have been used as low-cost adsorbents for the treatment of dye-containing wastewaters [2,4,7,8,9,12,16,20].

The raspberry (*Rubus idaeus*) is a fruit-bearing shrub that grows spontaneously in temperate countries in Europe, Asia, and North America. It is also widely cultivated in these regions, its fruits being very popular due to their sweet taste and high nutritional content. The fruits are used in the food, pharmaceutical, and cosmetic industries because they contain organic acids, sugar, vitamins (A, B1, B2, B5, B6, C, E), flavonoids, tannins, phenolic acids, carotene, cyanine, salicylic acid, calcium, magnesium, potassium, phosphorus, iron, and copper. They are used to treat cardiovascular and degenerative diseases, obesity, and cancer. Raspberry leaves, although used on a smaller scale than fruits, contain organic acids, flavonoids, tannins, and vitamin C and have the following many therapeutic properties: astringent, disinfectant, healing, antidiarrheal, antihemorrhagic, and hormone-regulating [24,25,26,27].

In this context, raspberry leaves represent a low-cost material, easily available in large quantities in nature in almost all regions of the world. Therefore, it can be a very useful adsorbent material.

This study offers a new approach to the dye adsorption on natural materials adsorbents, proposing for the first-time raspberry leaves for methylene blue removal from aqueous solutions. The research has a practical destination and is focused on the use of raspberry leaf-based material as an adsorbent in wastewater treatment. After an initial material characterization, the effect of the parameters that may influence the adsorption was investigated, and the optimal adsorption conditions were established using Taguchi experimental design. Equilibrium, kinetic, thermodynamic, and desorption investigations were also carried out.

## 2. Materials and Methods

The dried raspberry leaves were bought from a specialized company that collects and processes aromatic and medicinal plants, namely, StefMar (Ramnicu Valcea, Romania). Then, it was finely ground with an electric mill and then washed with distilled water to remove turbidity and color. In the last stage, the material was dried for 24 h, at 105 °C.

The SEM, FTIR, and color analysis were performed using a Quanta FEG 250 (FEI, Eindhoven, The Netherlands) microscope, a Shimadzu Prestige-21 FTIR (Shimadzu, Kyoto, Japan) spectrophotometer, and a Cary-Varian 300 Bio UV-VIS colorimeter (Varian Inc., Mulgrave, Australia) with integrating sphere and Spectralon standard, respectively. The SEM micrograph was acquired using a Large Field Detector (LFD) in low vacuum mode to avoid sample charging at a cathode voltage of 25 kV and a working distance of about 10.6 mm. The FTIR spectrum was recorded in the range 4000–450 cm^−1^ after the adsorbent sample was mixed thoroughly in a mortar with KBr and then put into a pellet-forming die. The color analysis was recorded for the D65 (natural light) under 10 observer angles.

The solid addition method [28] was used to determine the point of zero charges, pH_PZC_, where the net charge of the adsorbent surface is zero.

The adsorption experiments were carried out in 150 mL Erlenmeyer flasks where 50 mL of dye solution was stirred with the adsorbent material. Three independent replicates were performed for each experiment and throughout their duration the stirring intensity was kept constant. The pH was adjusted with dilute solutions of HCl and NaOH (0.1 N) while the ionic strength influence on adsorption process was studied using NaCl as background electrolyte. A Specord 200 PLUS UV-VIS (Analytik Jena, Jena, Germany) spectrophotometer (wavelength 664 nm) was used to determine the methylene blue concentration. The adsorption parameters and their range used in batch experiments were the following: pH (2–10), ionic strength (0–0.2 mol L^−1^), contact time (5–60 min), adsorbent dose (1–5 g L^−1^), initial dye concentration (20–250 mg L^−1^) and temperature (278–311 K).

The kinetic and equilibrium data were modeled using the non-linear equations of the pseudo-first-order, pseudo-second-order, Elovich, and Avrami kinetic models and the Langmuir, Freundlich, Temkin, and Sips isotherms, respectively [29,30,31,32,33]. Detailed information about these equations is presented in Supplementary Materials Table S1. This table also contains the calculation equations for the dye amounts adsorbed at equilibrium and the dye removal percentage. The higher determination coefficient (R^2^) value and the lower values for sum of square error (SSE), chi-square (χ^2^), and average relative error (ARE) were used to evaluate the suitability of kinetic models and adsorption isotherms [29]. The corresponding equations for these parameters are detailed in Supplementary Materials Table S2.

The values of specific thermodynamic parameters (standard Gibbs free energy change, standard enthalpy change, and standard entropy change) were calculated based on experimental data obtained at 278, 297, and 311 K according to equations described in Supplementary Materials Table S3 [29,34].

In order to improve the efficiency of dye removal, the optimal value of the parameters that influence the adsorption process was established using the Taguchi method (L27 orthogonal array experimental design). The results were assessed by ANOVA analysis, used to establish the percentage contribution of each parameter to the dye removal efficiency. Minitab 19 Software (version 19.1.1, Minitab LLC, State College, PA, USA) software was utilized to perform the required mathematical operations.

The desorption experiments were performed in batch system when dye-load adsorbent was mixed with three regenerating agents (0.1 N HCl, distilled water, and 0.1 N NaOH) at constant mixing intensity for 2 h.

## 3. Results and Discussion

### 3.1. Adsorbent Material Characterization

#### 3.1.1. FTIR Analysis

**Figure 1:**
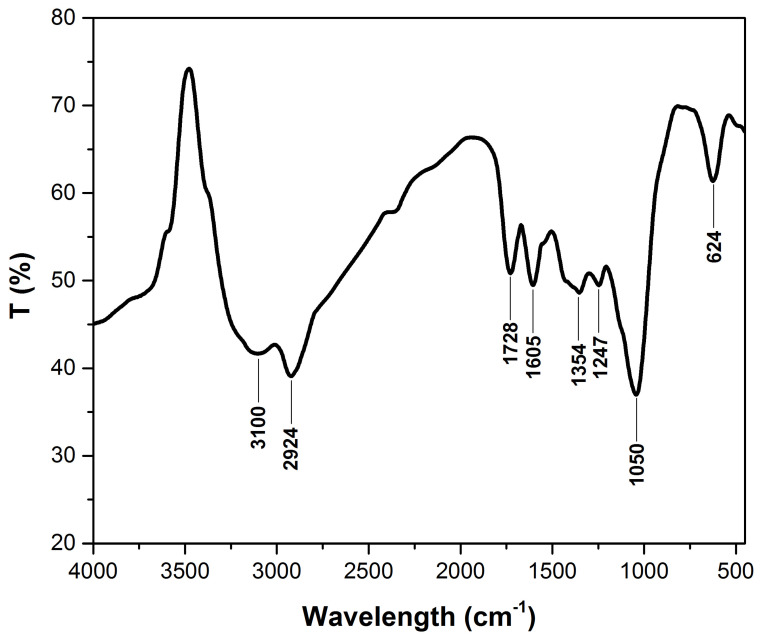
FTIR spectrum of raspberry leaves powder before and after adsorption.

Figure 1 shows the FTIR spectra of adsorbent materials. The registered peaks indicate that the main components of raspberry leaves are cellulose, hemicellulose, and lignin. The peaks between 3100 cm^−1^ and 2924 cm^−1^ can be assigned to the –OH groups and to the –CH_2_ groups of cellulose, respectively [35,36]. The C=O stretching vibration of the carboxylic group from lignin and hemicellulose can be observed at 1728 cm^−1^ [37]. The peak at 1605 cm^−1^ can be attributed to the aromatic skeletal and C=O stretch vibrations characteristic of lignin, and the peak at 1354 cm^−1^ can be assigned to –CH bending [35,38]. The C–O stretching vibration in lignin corresponds to the peak at 1247 cm^−1^. The band around 1050 cm^−1^ indicates the C–O–C stretching of cellulose, while the peak at 625 cm^−1^ is due to the bending modes of aromatic compounds [39,40,41].

Based on previous studies, the various functional groups from cellulose, hemi-cellulose, lignin, and pigments contained in leaves have a beneficial effect by generating active binding sites for dye adsorption [17,42,43].

#### 3.1.2. Color Analysis

**Figure 2:**
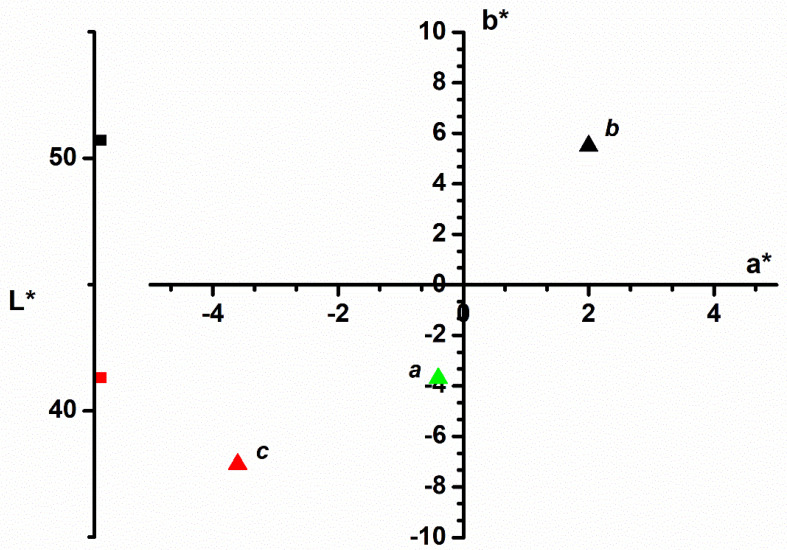
CIEL*a*b* color parameters of the following: *a*-methylene blue dye; *b*-raspberry leaves before adsorption process; *c*-raspberry leaves after adsorption process.

The color analysis of the adsorbent material was recorded for the D65 illuminant (natural daylight) and the standard 10° observer function, performed following the CIEL*a*b* color parameters (Figure 2). These parameters confirmed the change in the adsorbent color during the adsorption process.

The green color of fresh leaves may diminish by drying, but the vegetable wastes will maintain a residual color (Figure 2, point *b*). During the adsorption process, the color behavior shows that the dye color in the wastewater is transferred to the adsorption material. The luminosity of the adsorbent decreases, and the color parameters a* and b* show significant changes. Point *b*, characteristic of the initial color of the raspberry leaves, became point *c* after adsorption and moved into the color quarter of the methylene blue, initially described by point *a*.

#### 3.1.3. SEM Analysis

**Figure 3:**
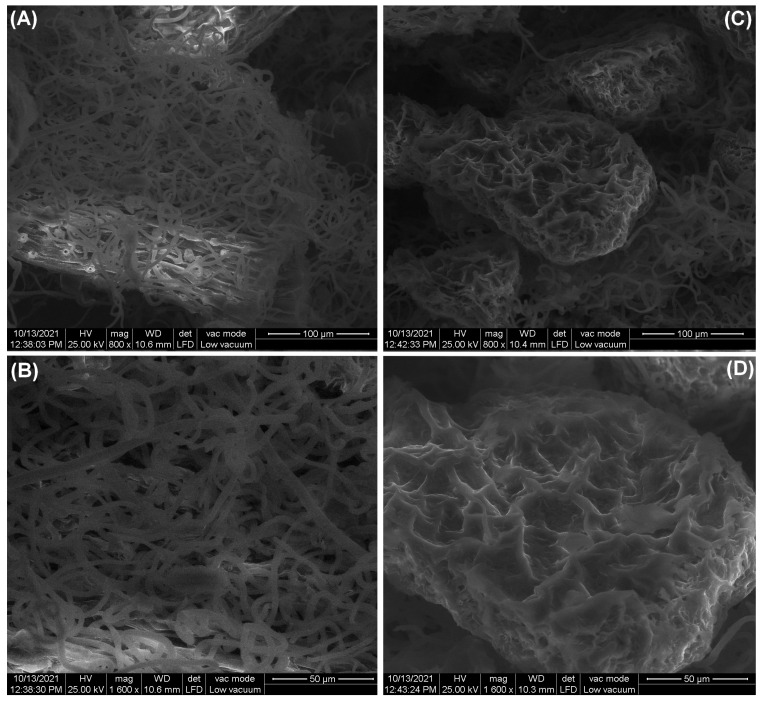
SEM images of adsorbent material surface before adsorption (**A**,**B**) and after adsorption (**C**,**D**) at different magnification.

The surface morphology of leaf powder, before and after adsorption, is illustrated in Figure 3. The images were acquired using the following two magnifications: 800× and 1600×. Initially, the surface is heterogeneous, having many irregularities, and is covered with trichomes. These are characteristic of raspberry leaves and have been highlighted in other articles in SEM images [25,44]. After adsorption, the surface becomes smoother and uniform, suggesting a dye molecular coating.

#### 3.1.4. Point of Zero Charge Determination

**Figure 4:**
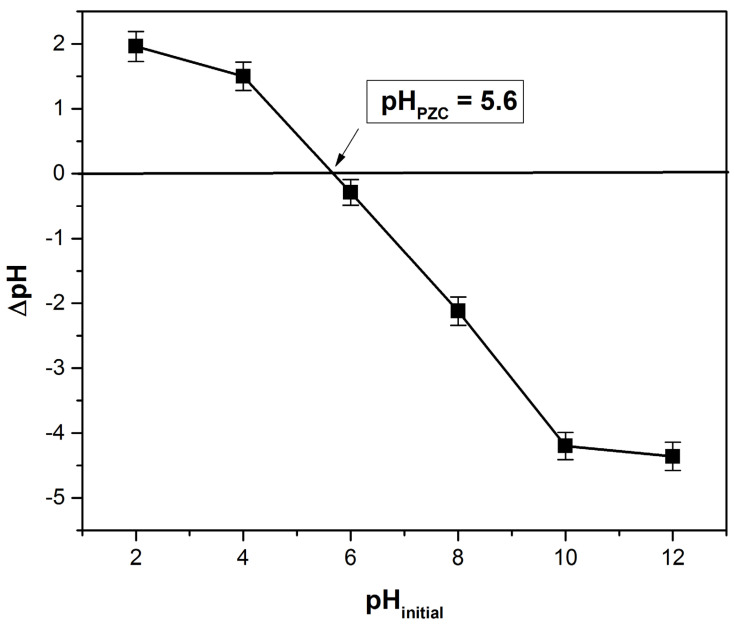
Determination of point of zero charge using the solid addition method.

pH_PZC_ has an important role in the adsorbent material’s surface characterization. The information given by this parameter suggests if an adsorbate will be adsorbed by an adsorbent based on the electric charge of its surface. If the solution pH is higher than pH_PZC_, the surface of the adsorbent is negatively charged, and the retention of positively charged species is favored. If the solution pH is lower than pH_PZC_, the surface of the adsorbent is positively charged, and the retention of negatively charged species is favored [7,8,17]. According to Figure 4, the determined pH_PZC_ value was 5.6. The same value was reported for poplar leaf powder [45], and close values were recorded for the following other similar adsorbents: 5.3 for *Magnolia grandiflora* leaves powder [46], 5.77 for *Syringa vulgaris* leaves powder [47], 6.3 for papaya leaf powder [48] and *Typha angustifolia* leaves powder [49].

### 3.2. Influence of pH and Ionic Strength

**Figure 5:**
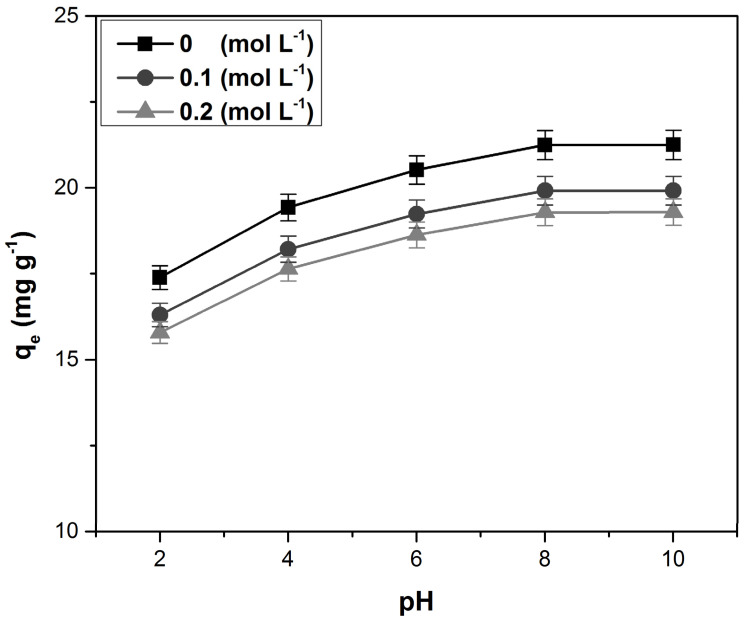
The dependence of adsorption capacity with the dye solution pH, at different ionic strengths.

The dependence of adsorption capacity on the dye solution pH at different ionic strengths is shown in Figure 5. Increasing the pH positively influences the adsorption capacity, while increasing the ionic strength has an unfavorable effect. For higher pH values than pH_PZC_ (5.6), the adsorbent material surface is negatively charged, and the electrostatic attraction favors the dye retention process [8,12,18,40,50].

Increasing ionic strength leads to worsening dye adsorption caused by the competition between dye cations and the other ions present in the solution in the process of occupying the available absorption sites on the adsorbent material surface [28,40,51].

The same variation of adsorption capacity with the pH and ionic strength was mentioned in other previous studies on similar adsorbents, such as the following: *Daucus carota* leaves powder [28], potato leaves powder [40], lilac tree leaves powder [47], pineapple leaf powder [52], and lotus leaf powder [53].

### 3.3. Kinetic Study

**Figure 6:**
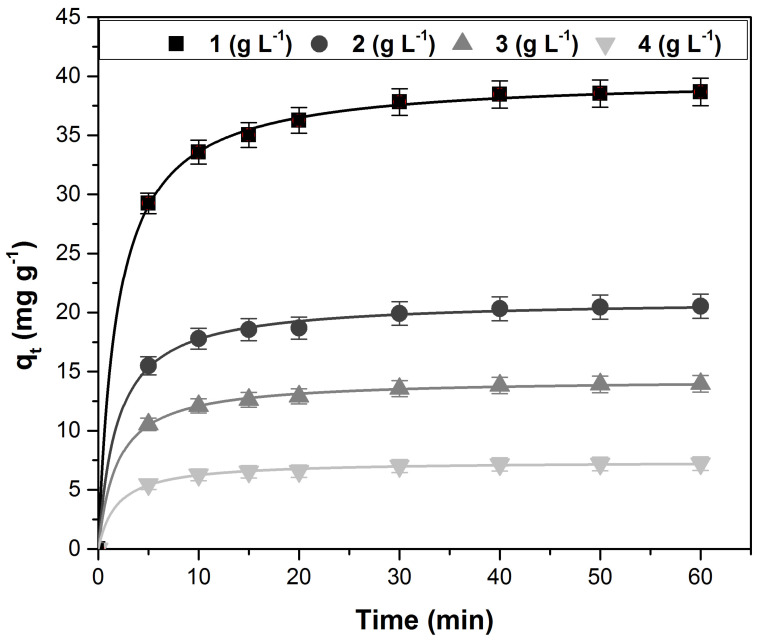
The variation of adsorption capacity with contact time at different doses of adsorbent material (fitted with pseudo-second-order kinetic model).

Figure 6 illustrates the variation of adsorption capacity with the contact time at different adsorbent doses. At the beginning of the process, the adsorbent surface has a high number of available adsorption sites; therefore, the adsorption rate is fast, and the adsorption capacity increases with the contact time [12,49,54]. As the adsorption sites are occupied by the dye molecules, the adsorption rate becomes slower until equilibrium is reached (after about 40 min), and thus the value of the adsorption capacity remains practically constant. The equilibrium times recorded in previous studies that used similar absorbent materials were the following: 20 min for papaya leaves [48] and *Humulus japonicas* leaves [55], 60 min for *Typha angustifolia* leaves [49] and pineapple leaf powder [52], 70 min for *Platanus orientalis* leaves [42], 100 min for *Ginkgo biloba* leaves [56] and pine tree leaves [57], 150 min for lotus leaves [53] and phoenix tree’s leaves [43], 180 min for poplar leaves [45], and 200 min for Miswak leaves [58].

Increasing the adsorbent dose leads to an increase in the number of places available for adsorption, but most of them remain unsaturated during the process and the adsorption capacity decreases. Moreover, the appearance of some aggregation or agglomeration of the adsorbent particles can contribute to a decrease in the adsorption capacity due to a decrease in the total adsorption surface and an increase in the diffusion path length [8,43,49].

In order to characterize the adsorption process from a kinetic point of view, several models were tested. The kinetic models, together with their constants and specific error parameters, are presented in Table 1. Analyzing the data in this table leads to the conclusion that the most suitable model to characterize the process is the pseudo-second-order kinetic model (higher value of R^2^ and lower values for SSE, χ^2^, and ARE) that was fitted in Figure 6.

### 3.4. Equilibrum Study

**Figure 7:**
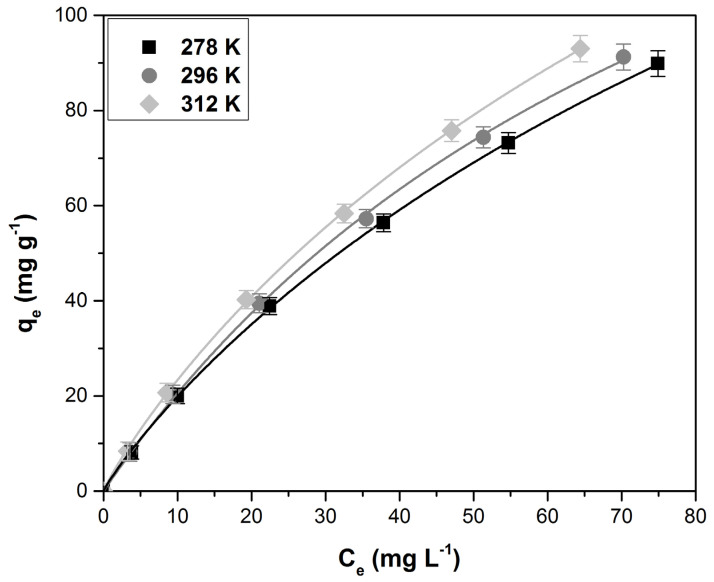
The fitted Sips isotherm curves at different temperatures.

Langmuir, Freundlich, Temkin, and Sips isotherms were tested in order to characterize the adsorption equilibrium. The constants of the tested isotherms and the corresponding error functions are presented in Table 2. The data show that the Sips isotherm best describes the process. This isotherm is a combined form of the Langmuir and Freundlich isotherms and is used to predict adsorption in heterogeneous systems and to avoid limiting the increasing concentration of adsorbate associated with the Freundlich model. At high concentrations of the adsorbate, one predicts a monolayer adsorption characteristic of the Langmuir isotherm, and at low concentrations, the adsorbate is reduced to the Freundlich isotherm [32,59]. The fitted Sips isotherm curves at different temperatures are presented in Figure 7. Increasing the temperature leads to a decrease in the solutions’ viscosity and, consequently, an increase in the dye molecules’ mobility, positively influencing the adsorption capacity and suggesting that the process is endothermic in nature [12,47].

By comparing the maximum adsorption capacity of raspberry leaf powder with other similar adsorbent materials reported in the literature, it can be seen that the investigated adsorbent is more effective than other adsorbents (Table 3).

### 3.5. Thermodynamic Parameters

The values of specific thermodynamic parameters (standard Gibbs free energy change, standard enthalpy changes, and standard entropy change) calculated based on experimental data obtained at 278, 297, and 311 K are summarized in Table 4. ΔG^0^ varied from −19.27 to −22.03 (kJ mol^−1^) when the temperature increased, and ΔH^0^ and ΔS^0^ had a positive value. Therefore, the adsorption is spontaneous, endothermic, and favorable, indicating the increasing randomness at the solid-solution interface during the process [8,20,53]. Similar behavior was reported by other researchers’ papers on the methylene blue removal from aqueous solutions using adsorbents based on plant leaves [8,46,47,56,60,67].

When ΔG^0^ values ranged between −20 (kJ mol^−1^) and 0 (kJ mol^−1^), physisorption is involved in the process, and when ΔG^0^ values ranged between −20 (kJ mol^−1^) and −80 (kJ mol^−1^), both physical and chemical adsorption are involved [47,56,68,69]. The standard Gibbs free energy change values suggest that physisorption is involved in the process, but a small chemical effect appears that may enhance the process. The standard enthalpy change value lower than 20 (kJ mol^−1^) confirms the physical adsorption and indicates that van der Waals interactions have an important role in the process [70,71,72].

### 3.6. Adsorption Parameters Optimization

**Figure 8:**
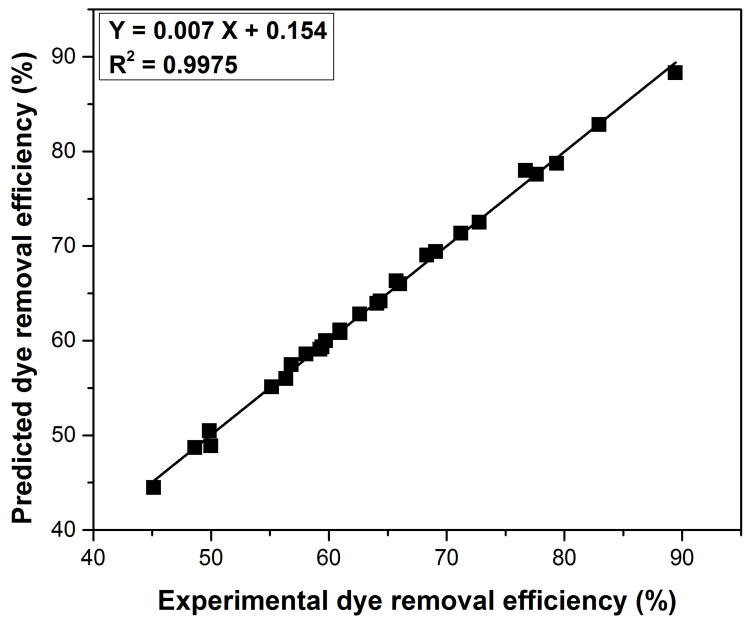
Correlation of experimental and predicted dye removal efficiency.

The present paper uses the Taguchi method as the most suitable optimization algorithm to understand the variable process parameters’ effects on the adsorption efficiency. Compared with other methods, the ranking approach offered by this method allows better visualization of the optimal conditions and requires less experimental data [73,74]. The Taguchi method uses a minimized number of experiments and converts the obtained experimental results into a signal-to-noise (S/N) ratio, which describes the level of dispersion and degree of optimization [47,73,74]. The L27 orthogonal array used in the Taguchi design was made using six controllable factors at three levels. Table 5 shows these factors and the effect of the 27 experiments, performed according to the orthogonal array, on methylene blue removal efficiency and the signal to noise (S/N) ratios. The results evaluation was achieved using the “larger is the better” option for the (S/N) ratio. The controllable factors’ significance was established by the S/N ratio rank and is presented in Table 6 together with their rank. The contact time was the factor having the highest influence on the process, while the temperature was the factor with the lowest influence. The optimal conditions for methylene blue removal were the following: pH 10, ionic strength 0.0 (mol L^−1^), contact time 40 (min), adsorbent dose of 5 (g L^−1^), initial dye concentration of 20 (mg g^−1^), and temperature of 312 K. The ANOVA analysis validated the order of controllable factor influence obtained with the Taguchi method and established the percentage contribution of each factor (Table 6). The correlation between the experimental results on the dye removal efficiency with the predicted values by the Taguchi method shows that the prediction accuracy is very good, with the value of R^2^ being close to 1 (Figure 8).

### 3.7. Desorption Study

Three desorbing agents (0.1 N HCl, distilled water, and 0.1 N NaOH) were used to study the feasibility of the adsorbent regeneration after dye adsorption. The best results were obtained when HCl was used (Table 7). The regenerated adsorbent was used again for dye adsorption in the optimal conditions established by the Taguchi method. The dye removal efficiency was only 49.6%, and therefore the adsorbent regeneration is not technically and economically justified. This is not a big disadvantage because the raspberry leaves represent a low-cost material that is easily available in large quantities in nature. In addition, based on the combustion properties of plant leaves, the exhausted adsorbent results after adsorption can be incinerated. Based on the high gas amount generated by combustion, the exhausted adsorbent can be used as a porogenous precursor for cellular glasses.

## 4. Conclusions

The adsorption study presented in the paper is intended to have a practical destination and is focused on the use of raspberry leaf-based material as an adsorbent for the removal of dyes from aqueous effluents, with applications in wastewater treatment. The effect of parameters that can influence the adsorption process in industrial practice was the main objective of the study, along with their optimization.

The FTIR spectra show various functional groups from cellulose, hemi-cellulose, and lignin contained by leaves that have a positive effect by providing active binding sites for dye adsorption.

The color analysis of the adsorbent material recorded before and after adsorption suggests the dye retention on the leave powder. This idea is confirmed by SEM images that illustrate the significant changes in the adsorbent surface texture and morphology.

pH_PZC_ is an important parameter for the adsorbent material’s surface characterization, suggesting whether an adsorbate will be adsorbed by an adsorbent based on the electric charge of its surface. The determined pH_PZC_ = 5.6, a value close to other similar adsorbents reported in the literature.

The adsorption capacity of the adsorbent material is positively influenced by the increase in pH, contact time, temperature, adsorbent dose, and the decrease in the initial dye concentration and ionic strength.

The equilibrium and kinetic studies reveal that the Sips isotherm and pseudo-second-order kinetic model best describe the process. The maximum value of the adsorption capacity was 244.6 (mg g^−1^), which is higher compared to other plant-leaf-based adsorbents.

The thermodynamic parameter values suggest a spontaneous, favorable, and endothermic process involving physisorption as the main mechanism.

The Taguchi approach and the ANOVA analysis indicate that contact time was the factor having the higher influence on the process (47.26%), followed by pH (27.10%), initial dye concentration (11.65%), adsorbent dose (7.35%), ionic strength (5.92%), and temperature (0.73%).

The low cost, abundance, and ease of finding in nature along with a good adsorption capacity make the proposed material a suitable and effective adsorbent in removing methylene blue dye from water.

## Figures and Tables

**Table 1:** The kinetic models’ constants and the corresponding error functions.

Kinetic Model	Parameters	Adsorbent Dose (g L^−1^)			
		1	2	3	4
Pseudo-first-order	k_1_ (min^−1^)	0.275 ± 0.035	0.279 ± 0.047	0.277 ± 0.054	0.279 ± 0.051
	q_e,calc_ (mg g^−1^)	37.48 ± 0.82	19.76 ± 0.29	13.47 ± 0.31	6.95 ± 0.27
	R^2^	0.9901	0.9876	0.9891	0.9876
	χ^2^	0.3429	0.2235	0.1347	0.0786
	SSE	12.11	4.23	1.72	0.52
	ARE (%)	13.96	14.34	14.14	14.36
Pseudo-second-order	k_2_ (min^−1^)	0.013 ± 0.005	0.025 ± 0.010	0.037 ± 0.011	0.071 ± 0.026
	q_e,calc_ (g mg^−1^ min^−1^)	39.92 ± 0.89	21.07 ± 0.74	14.36 ± 0.71	7.40 ± 0.32
	R^2^	0.9996	0.9986	0.9993	0.9986
	χ^2^	0.0110	0.0251	0.0078	0.0087
	SSE	0.38	0.47	0.10	0.05
	ARE (%)	0.50	0.99	0.72	0.99
Elovich	a (g mg^−1^)	0.267 ± 0.052	0.506 ± 0.061	0.741 ± 0.057	1.447 ± 0.075
	b (mg g^−1^ min^−1^)	2508 ± 153	1328 ± 125	895 ± 96	895 ± 96
	R^2^	0.9969	0.9973	0.9973	0.9974
	χ^2^	0.1336	0.0637	0.0436	0.0227
	SSE	3.72	0.90	0.42	0.11
	ARE (%)	12.68	12.57	12.58	12.52
Avrami	k_AV_ (min^−1^)	0.633 ± 0.046	0.638 ± 0.057	0.635 ± 0.062	0.638 ± 0.073
	q_AV_ (mg g^−1^)	37.48 ± 0.51	19.76 ± 0.41	13.47 ± 0.45	6.95 ± 0.26
	n_AV_	0.435	0.437	0.436	0.437
	R^2^	0.9901	0.9876	0.9891	0.9876
	χ^2^	0.3419	0.2231	0.1344	0.0784
	SSE	12.11	4.23	1.72	0.52
	ARE (%)	13.96	14.34	14.14	14.34

**Table 2:** The adsorption isotherms models constants and the corresponding error functions.

Isotherm Model	Parameters	Value		
		278 K	297 K	311 K
Langmuir	K_L_ (L mg^−1^)	0.011 ± 0.001	0.012 ± 0.001	0.013 ± 0.002
	q_max_ (mg g^−1^)	186.08 ± 7.25	188.96 ± 6.12	192.58 ± 6.73
	R^2^	0.9997	0.9997	0.9997
	χ^2^	0.0321	0.0326	0.0332
	SSE	1.18	1.21	1.26
	ARE (%)	1.43	1.44	1.43
Freundlich	K_f_ (mg g^−1^)	3.54 ± 0.61	3.78 ± 0.52	4.11 ± 0.71
	1/n	0.76 ± 0.07	0.75 ± 0.05	0.76 ± 0.04
	R^2^	0.9988	0.9988	0.9988
	χ^2^	0.3367	0.3419	0.3483
	SSE	5.23	5.40	5.60
	ARE (%)	4.74	4.74	4.74
Temkin	K_T_ (L mg^−1^)	0.291 ± 0.043	0.311 ± 0.057	0.339 ± 0.052
	b (kJ g^−1^)	102.22 ± 7.49	100.67 ± 5.71	98.77 ± 6.18
	R^2^	0.9632	0.9632	0.9633
	χ^2^	16.41	16.67	17.00
	SSE	149.75	154.42	160.39
	ARE (%)	57.29	57.29	58.15
Sips	Q_sat_ (mg g^−1^)	235.5 ± 8.45	239.1 ± 6.27	244.6 ± 7.64
	K_S_ (L mg^−1^)	0.010 ± 0.001	0.011 ± 0.002	0.012 ± 0.002
	n	0.9326	0.9326	0.9319
	R^2^	0.9998	0.9998	0.9998
	χ^2^	0.0242	0.0246	0.0254
	SSE	0.56	0.58	0.60
	ARE (%)	1.42	1.42	1.43

**Table 3:** The maximum adsorption capacities for different adsorbent materials based on plant leaves.

Adsorbent	Maximum Adsorption Capacity (mg g^−1^)	Reference
*Ficcus Palmata* leaves	6.89	[60]
*Alchemilla Vulgaris* leaves	45.66	[61]
*Ginkgo biloba* leaves	48.07	[56]
*Salix babylonica* leaves	60.9	[17]
*Daucus carota* leaves powder	66.5	[28]
Phoenix tree’s leaves	80.90	[43]
*Acer* tree leaves	97.07	[62]
*Elaeis guineensis* leaves	103.00	[20]
*Typha angustifolia* leaves	106.75	[49]
Banana leaves	109.90	[63]
*Cocos nucifera* leaf	112.35	[64]
*Platanus orientalis* leaf powder	114.90	[42]
Pine Tree leaves	126.58	[57]
Poplar leaf	135.40	[45]
*Humulus japonicas* leaves	145.56	[55]
*Magnolia grandiflora* leaves	149.25	[46]
*Magnolia denudate* leaves	185.19	[46]
Gulmohar leaf	186.24	[65]
*Syringa vulgaris* leaves	188.2	[47]
Miswak leaves	200	[58]
Lotus leaf	221.7	[53]
*Michelia figo* leaves	238.1	[46]
**Raspberry *(Rubus idaeus)* leaves**	**244.6**	**This study**
Guava leaf	295.04	[66]
*Buxus sepmervirens* leaves	384.61	[67]
Papaya leaf	512.55	[48]

**Table 4:** The thermodynamic parameters for the dye adsorption on raspberry leaf powder.

ΔG^0^ (kJ mol^−1^)			ΔH^0^ (kJ mol^−1^)	ΔS^0^ (J mol^−1^ K^−1^)
278 K	297 K	311 K		
−19.27	−20.68	−22.03	0.38	9.70

**Table 5:** The L27 orthogonal array used in the Taguchi design.

pH	Ionic Strength (mol L^−1^)	Time (min)	Adsorbent Dose (g L^−1^)	Initial Dye Concentration (mg g^−1^)	Temperature (K)	Dye Removal Efficiency (%)	S/N Ratio
2	0	5	1	20	278	49.87	33.95
2	0	5	1	100	297	48.62	33.73
2	0	5	1	250	311	45.12	33.08
2	0.1	20	3	20	278	60.96	35.70
2	0.1	20	3	100	297	59.42	35.47
2	0.1	20	3	250	311	55.15	34.83
2	0.2	40	5	20	278	66.03	36.39
2	0.2	40	5	100	297	64.37	36.17
2	0.2	40	5	250	311	59.74	35.52
6	0	20	5	20	297	79.35	37.99
6	0	20	5	100	311	77.64	37.80
6	0	20	5	250	278	68.30	36.68
6	0.1	40	1	20	297	72.78	37.24
6	0.1	40	1	100	311	71.21	37.05
6	0.1	40	1	250	278	62.65	35.93
6	0.2	5	3	20	297	58.07	35.28
6	0.2	5	3	100	311	56.82	35.09
6	0.2	5	3	250	278	49.99	33.97
10	0	40	3	20	311	89.43	39.03
10	0	40	3	100	278	82.95	38.37
10	0	40	3	250	297	76.70	37.69
10	0.1	5	5	20	311	65.73	36.35
10	0.1	5	5	100	278	60.97	35.70
10	0.1	5	5	250	297	56.37	35.02
10	0.2	20	1	20	311	69.08	36.78
10	0.2	20	1	100	278	64.08	36.13
10	0.2	20	1	250	297	59.25	35.45

**Table 6:** Response table for signal-to-noise S/N ratios (larger is better).

Level	pH	Ionic Strength	Time	Adsorbent Dose	Initial Concentration	Temperature
1	34.99	36.49	34.69	35.49	36.53	35.87
2	36.34	35.92	36.32	36.16	36.17	36.01
3	36.73	35.65	37.05	36.41	35.36	36.17
Delta	1.74	0.84	2.36	0.92	1.17	0.30
Rank	2	5	1	4	3	6
Contribution (%)	27.10	5.92	47.26	7.35	11.65	0.73

**Table 7:** The efficiencies of the used desorbing agents.

Desorbing Agent	Desorption Efficiencies (%)
HCl	77.63 ± 2.51
Distilled water	8.08 ± 0.25
NaOH	36.95 ± 1.78

## Data Availability

All the experimental data obtained are presented, in the form of table and/or figure, in the article and in the Supplementary Materials.

## Supplementary Materials

The following are available online at www.mdpi.com/article/10.3390/polym14101966/s1, Table S1: The non-linear equations of the pseudo-first-order, pseudo-second-order, Elovich, Avrami kinetic models, and the Langmuir, Freundlich, Temkin, and Sips isotherms, Table S2: The corresponding equations for determination coefficient (R^2^), sum of square error (SSE), chi-square (χ^2^), and average relative error (ARE), Table S3: The equations of specific thermodynamic parameters (standard Gibbs free energy change, standard enthalpy change, and standard entropy change).
