# Supplementary material for: Removal of Methylene Blue from Aqueous Solutions Using a New Natural Lignocellulosic Adsorbent—Raspberry (Rubus idaeus) Leaves Powder
Source: Polymers (Basel). 2022 May 11;14(10):1966. doi: 10.3390/polym14101966 (PMC9143437; doi:10.3390/polym14101966)
Supplement: Supplementary file 1 [file polymers-14-01966-s001.zip › polymers-1710432-supplementary.pdf]

polymers

MDPI

Supplementary materials

Removal of methylene blue from aqueous solutions using a new natural lignocellulosic adsorbent - raspberry (*Rubus idaeus*) leaves powder

Giannin Mosoarca [1], Simona Popa [1*], Cosmin Vancea [1*], Mircea Dan [1*] and Sorina Boran [1]

[1] Politehnica University Timisoara, Faculty of Industrial Chemistry and Environmental Engineering, Bd. V. Parvan No. 6, 300223, Timisoara, Romania; giannin.mosoarca@upt.ro; sorina.boran@upt.ro

* Correspondence: (G.M.); simona.popa@upt.ro (S.P.); cosmin.vancea@upt.ro (C.V.); mircea.dan@upt.ro (M.D.); Tel.: +40-256404212 (S.P.); +40-256404194 (C.V.); +40-256404176 (M.D.)

Table S1. The non-linear equations of the pseudo-first order, pseudo-second order, Elovich, Avrami kinetic models and the Langmuir, Freundlich, Temkin, Sips isotherms.

Kinetic and isotherm models	Equation
Pseudo-first-order kinetic model	$q_t = q_e(1 - exp^{-k_1 \cdot t})$
Pseudo-second-order kinetic model	$q_t = \frac{k_2 \cdot t \cdot q_e^2}{1 + k_2 \cdot t \cdot q_e}$
Elovich kinetic model	$q_t = \frac{1}{a} ln(1 + a \cdot b \cdot t)$
Avrami kinetic model	$q_t = q_{AV}[1 - exp(-k_{AV} \cdot t)^{n_{AV}}]$
Langmuir isotherm	$q_e = \frac{q_m \cdot K_L \cdot C_e}{1 + K_L \cdot C_e}$
Freundlich isotherm	$q_e = K_F \cdot C_e^{1/n_F}$
Temkin isotherm	$q_e = \frac{R \cdot T}{b} \cdot ln(K_T \cdot C_e)$
Sips isotherm	$q_e = \frac{Q_{sat} \cdot K_S \cdot C_e^n}{1 + K_S \cdot C_e^n}$

where: q_t is the dye amount adsorbed at time t; k_1, k_2 and k_{AV} are the rate constants of pseudo-first-order, pseudo-second-order and Avrami kinetic models; q_e and q_{AV} are the theoretical values for the adsorption capacity; a is the desorption constant of Elovich model; b is the initial velocity; n_{AV} is a fractional exponent; q_m and Q_{sat} are the maximum absorption capacities; K_L, K_F, K_T and K_S are the Langmuir, Freundlich, Temkin and Sips isotherms constants; $1/n_F$ is an empirical constant indicating the intensity of adsorption; R is the universal gas constant; T is the absolute temperature; b is Temkin constant which related to the adsorption heat; n is Sips isotherm exponent.

Table S2. The corresponding equations for determination coefficient (R^2), sum of square error (SSE), chi-square (χ^2) and average relative error (ARE).

Function name	Equation
Determination coefficient	$R^2 = 1 - \frac{\sum_{i=1}^{n}\left(y_{i,exp} - y_{i,mod}\right)^2}{\sum_{i=1}^{n}\left(y_{i,exp} - \overline{y_{i,exp}}\right)^2}$
Sum of square error	$SSE = \sum_{i=1}^{n}\left(y_{i,exp} - y_{i,mod}\right)^2$
Chi-square	$\chi^2 = \sum_{i=1}^{n}\frac{\left(y_{i,exp} - y_{i,mod}\right)^2}{y_{i,mod}}$
Average relative error	$ARE = \frac{100}{n}\sum_{i=1}^{n}\left\lvert\frac{y_{i,exp} - y_{i,mod}}{y_{i,mod}}\right\rvert$

where: $y_{i,exp}$ is the experimental value; $y_{i,mod}$ is the modeled value; $\overline{y_{i,exp}}$ is the mean values and n is the total amount of information.

Table S3: The equations of specific thermodynamic parameters (standard Gibbs free energy change, standard enthalpy change and standard entropy change).

Thermodynamic parameters	Equation
Standard Gibbs free energy change	$\Delta G^0 = -RT \ln K_L$
Standard enthalpy change<br>Standard entropy change	$\ln K_L = \frac{\Delta S^0}{R} - \frac{\Delta H^0}{RT}$

where: R is the universal gas constant; K_L is the Langmuir constant and T is the absolute temperature.
